# Peculiar Presentation of COVID-19: A Case Report of Concurrent Stroke and Guillain–Barré Syndrome

**DOI:** 10.1155/2021/8824512

**Published:** 2021-02-15

**Authors:** Behnaz Ansari, Helia Hemasian

**Affiliations:** Isfahan Neuroscience Research Center, Department of Neurology, Al Zahra Research Institute, Isfahan University of Medical Sciences, Isfahan, Iran

## Abstract

**Background:**

Coronavirus disease 2019 (COVID-19) is a newly recognized infectious disease that has turned into a pandemic. There are few studies reporting Guillain–Barré syndrome (GBS) and stroke separately associated with COVID-19. In this study, we report an unusual case of COVID-19 with stroke and GBS concurrently. *Case Report*. A 59-year-old woman presented with left-sided weakness of two weeks' duration followed by right-sided weakness and foot paresthesia. She also complained of cough, myalgia, and respiratory distress of three weeks' duration. On examination, the patient had respiratory distress. The limb examination revealed asymmetric weakness. All limb reflexes were absent. Pinprick sensation was impaired. The chest CT scan and PCR of nasopharyngeal swab confirmed the diagnosis of COVID-19. Further evaluation revealed acute cerebral infarction and GBS. Consequently, the patient was treated by plasmapheresis, and her symptoms partially improved.

**Conclusion:**

According to reports, 36.4% of COVID-19 cases display neurological complications. The neurological manifestations of the disease can involve both the central and peripheral nervous systems. Previously, a few cases of GBS and cerebrovascular disease have been reported in association with COVID-19 separately, while in the present case, CNS and PNS involvement occurred concurrently. It is hypothesized that this concurrence is related to the imbalance of the systemic inflammatory responses and blood vessel autonomous dysfunction.

## 1. Introduction

Coronavirus disease 2019 (COVID-19), originated in China, is rapidly spreading throughout the world. This is an infectious disease caused by the severe acute respiratory syndrome coronavirus 2 (SARS-CoV-2). Evidence increasingly shows that SARS-CoV-2 is not always confined to the respiratory system and can induce neurological disorders as well. Both central and peripheral nervous system disorders have been reported following COVID-19 [1]. Acute clinical neurological syndromes of COVID-19 fall into three main categories: stroke, Guillain–Barré syndrome (GBS), and meningoencephalitis, encephalopathy, or encephalitis [2]. About one-third of COVID-19 patients develop neurological symptoms. Damage to the CNS or PNS could be caused by the virus directly or by the body's adaptive immune responses to the infection [1].

There are several case reports of stroke and GBS separately in association with COVID-19. In this study, we report an unusual case of COVID-19 with CNS and PNS involvement concurrently.

## 2. Case Report

A 59-year-old woman with a past medical history of hypertension and stroke presented to the emergency department with left-sided weakness of two weeks' duration followed by right-sided weakness and foot paresthesia. She also complained of cough, myalgia, and respiratory distress of three weeks' duration and reported dizziness and falling down 2 days before admission. She had no bladder symptoms of note.

On examination, the patient was afebrile. Oxygen saturation was 90% on air and respiratory rate was 26 breaths/min. The limb examination revealed an asymmetric weakness of −3/5 in the right upper and lower limbs and 2/5 on the left side. All limb reflexes were absent. No Babinski sign was found. Pinprick sensation was impaired to the ankle on both sides, with impaired vibration and position senses in the left lower limb. Cranial nerves were intact.

On admission, white blood cell count was 12300 cells per microliter (neutrophils: 80.9%; lymphocytes: 12.7%). The renal profile, electrolytes, FBS, HbA1C, serum angiotensin converting enzyme (ACE), B12, and thyroid and clotting functions were all within the normal range. Vasculitis, HIV, and hepatitis B and C tests were negative.

CRP was mildly positive, and D-dimer and IL-6 levels had not increased.

**Figure 1:**
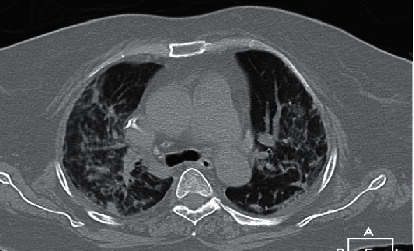
Chest computed tomography on admission showed multilobar ground-glass opacity with reticulation and consolidation in the center and periphery of both lungs.

**Figure 2:**
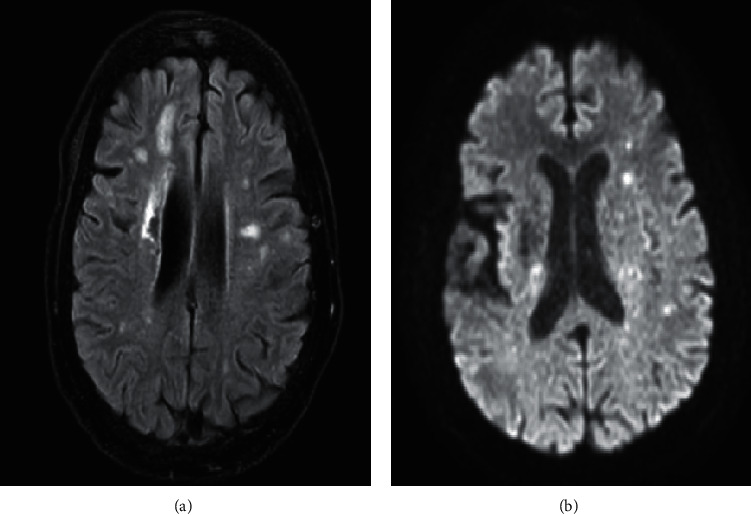
Magnetic resonance imaging. (a) Fluid-attenuated inversion recovery (FLAIR) and (b) diffusion-weighted sequence (DWI) revealed acute infarct in the left centrum semiovale and old infarct on the right side.

The patient's chest CT scan on admission (Figure 1) showed multilobar ground-glass opacity with reticulation and consolidation in the center and periphery of both lungs. The nasopharyngeal swab PCR test was positive for SARS-CoV-2. Because of the asymmetric and unusual presentation, brain and spinal cord imaging were performed. In the brain MRI, acute infarct in the left centrum semiovale, as well as an old infarct on the right side, was detected (Figure 2). Cardiac and carotid investigation results were within the normal limits. No obvious intracranial stenosis was detected in the transcranial duplex study.

The patient was admitted with suspected GBS secondary to COVID-19. Lumbar puncture was tried but failed. Nerve conduction studies were performed and revealed reduced amplitude and velocity in all tested sensory nerves in the upper limbs, increased latency in both median nerves, and absence of response in the lower limbs. Reduced amplitude and conduction velocity with an asymmetric pattern (left > right) and prolonged distal latency in both median and left ulnar motor nerves, as well as the absence of response in the lower limbs, were noticed. No *F* waves could be elicited from either of the median nerves and the left ulnar nerve (Table 1). In electromyography, no spontaneous activity was detected; moreover, reduced recruitment in both tibialis anterior and left gastrocnemius muscles and a normal pattern in the upper limbs were observed. These results meet the electrodiagnostic criteria for acute inflammatory demyelinating polyneuropathy, and consequently, treatment was started with plasma exchange (PLEX), ASA (80 mg/day), and enoxaparin (1 mg/kg). In follow-up, the patient's weakness obviously improved with PLEX. Written consent was obtained from the patient for publishing the contents of this study.

## 3. Discussion

COVID-19 presents with a variety of symptoms, ranging from asymptomatic to severe, rapid multiorgan dysfunction and death. Although the predominant clinical presentation is respiratory disease, neurological manifestations are being increasingly recognized.

In an analysis of 214 cases of COVID-19 in Wuhan, China, 36.4% had neurological complications [3]. These manifestations may be direct effects of the virus on the nervous system, parainfectious or postinfectious immune-mediated disease, or neurological complications of the systemic effects of COVID-19 [1]. The neurological manifestations of this disease involve either the central or peripheral nervous system. Surprisingly, in the case presented here, CNS and PNS involvement occurred concurrently.

One of the peripheral nervous system complications of COVID-19 is the Guillain–Barré syndrome; also, one of its central nervous complications is cerebrovascular accident. A number of cases of GBS have been reported in patients with COVID-19 [4–10]. GBS is an infrequent complication of COVID-19. Short interval between viral infection and the onset of weakness, as in the present case, suggests a parainfectious rather than a postinfectious nature for it [5]. One possible immunological explanation for this phenomenon is the cytokine release syndrome (CRS) in response to COVID-19 [11, 12], which can produce extensive tissue damage, including in the peripheral nervous system [13].

Acute cerebrovascular disease is emerging as a significant complication of COVID-19, with cohort studies reporting stroke in 2–6% of hospitalized patients with the disease [1]. Cerebrovascular disease in COVID-19 might be due to abnormalities in the coagulation cascade; SARS-CoV-2 can cause damage to endothelial cells and activate inflammatory and thrombotic pathways. Hence, cytokines have been implicated in the pathogenesis of cerebrovascular disease in COVID-19 through a systemic inflammatory response syndrome and induction of a proinflammatory condition [14]. There are reports of various types of stroke in COVID-19 [1], including intracerebral hemorrhage [15, 16] and cerebral sinus venous thrombosis [17, 18], but not of watershed infarct.

Both GBS and cerebral stroke are critical neurological diseases, and their clinical symptoms may be obscured by each other. However, the exact mechanism of their relationship is not known. Wu et al. discussed patients with both hemorrhagic infarct and GBS and attributed this concurrence to the imbalance of systemic inflammatory responses, blood vessel autonomous dysfunction, and the use of intravenous immunoglobulin (IVIg) [19].

Rarely, cases of cerebral infarction have been reported after the management of GBS with IVIg [20]. These cases are believed to be due to hyperviscosity, thromboembolism, vasculitis, or cerebral vasospasm; however, our patient had not received IVIg. As mentioned, she experienced hypotension and falling prior to admission. Furthermore, dysautonomia due to GBS results in fluctuations of hemodynamic parameters, which could be the cause of cerebral infarct of watershed areas in our patient. Another possible cause is the imbalance of inflammatory responses due to COVID-19.

## 4. Conclusion

Neurologic complications of COVID-19 vary from a mild headache to severe disorders, and as discussed, it is not inconceivable that they could involve the CNS and PNS concurrently, in which case, clinical symptoms of the two involvements may cover each other, leading to confusion in diagnosis. Generally, limited case reports only suggest a possible association, and more cases with epidemiological data are necessary to support a causal relationship.

## Figures and Tables

**Table 1:** Results of the neurophysiologic study.

*Motor nerve conduction*	Distal latency (ms)	Amplitude (mV)	Conduction velocity (m/s)	*F* waves

Median nerve				
Wrist-abductor pollicis brevis	*R* = 5.42; *L* = 4.69 (n.v. < 4.2)	*R* = 2.1; *L* = 1.2 (n.v. > 4)	*R* = 35; *L* = 38 (n.v. > 50)	*R* = *A*; *L* = *A*
Antecubital fossa-wrist	*R* = 10.10; *L* = 9.27	*R* = 2.1; *L* = 1.2		
Ulnar nerve				
Wrist-abductor digiti minimi	*R* = 3.7; *L* = 6.77 (n.v. < 3.2)	*R* = 4.3; *L* = 0.5 (n.v. > 6)	*R* = 35; *L* = *A* (n.v. > 50)	*R* = 24; *L* = *A*
Below elbow-wrist	*R* = 6.61; *L* = *a*	*R* = 3.3; *L* = *a*		
Tibial nerve				
Medial malleolus-abductor hallucis brevis	*R* = *a*; *L* = *a* (n.v. < 5)	*R* = *a*; *L* = *a* (n.v. > 4)	*R* = A; *L* = A (n.v. > 40)	*R* = A; *L* = A
Popliteal fossa-medial malleolus	*R* = *a*; *L* = *a*	*R* = *a*; *L* = *a*		
Peroneal nerve				
Ankle-extensor digitorum brevis	*R* = *a*; *L* = *a* (n.v. < 6.5)	*R* = *a*; *L* = *a* (n.v. > 2)	*R* = *A*; *L* = *A* (n.v. > 40)	*R* = *A*; *L* = *A*
Below fibula-ankle	*R* = *a*; *L* = *a*	*R* = *a*; *L* = *a*		

*Antidromic sensory nerve conduction studies*	
Median nerve digit 2-wrist	*R* = 3.80; *L* = 3.7 (n.v. < 3.5)	*R* = 8.9; *L* = 10.7 (n.v. > 20)	*R* = 34; *L* = 44 (n.v. > 45)	
Ulnar nerve digit 5-wrist	*R* = 2.60; *L* = 2.50 (n.v. < 3.2)	*R* = 15.1; *L* = 9.2 (n.v. > 17)	*R* = 42, *L* = 44 (n.v. > 45)	
Sural nerve posterior ankle-calf	*R* = *A*; *L* = *A* (n.v. < 4.4)	*R* = *A*; *L* = *A* (n.v. > 6)	*R* = *A*; *L* = *A* (n.v. > 40)	

*R*, right; *L*, left; n.v., normal values adjusted for age; *A*, absent.
